# Environmental Factors Related to Pulmonary Tuberculosis in HIV-Infected Patients in the Combined Antiretroviral Therapy (cART) Era

**DOI:** 10.1371/journal.pone.0165944

**Published:** 2016-11-03

**Authors:** Alejandro Álvaro-Meca, Asuncion Díaz, Javier de Miguel Díez, Rosa Resino, Salvador Resino

**Affiliations:** 1 Department of Preventive Medicine & Public Health, Rey Juan Carlos University, Madrid, Spain; 2 Unit of HIV Surveillance and Behavioural Monitoring. National Center of Epidemiology, Institute of Health Carlos III, Madrid, Spain; 3 Network of Biomedical Research Centers Epidemiology and Public Health (Centro de Investigacion Biomédica en Red de Epidemiología y Salud Pública (CIBERESP)), Madrid, Spain; 4 Pneumology Service, Hospital General Universitario Gregorio Marañón. Universidad Complutense de Madrid, Madrid, Spain; 5 Department of Human Geography, Faculty of Geography and History, Complutense University of Madrid. Madrid, Spain; 6 Unit of Viral Infection and Immunity, National Center for Microbiology, Institute of Health Carlos III, Majadahonda. Madrid, Spain; Universidade Nova de Lisboa Instituto de Higiene e Medicina Tropical, PORTUGAL

## Abstract

The aim of our study was to evaluate the seasonal variations and whether short-term exposure to environmental risk factors, such as climate and air pollution, is associated with PTB-related hospital admissions in human immunodeficiency virus (HIV)-infected patients in Spain during the era of combined antiretroviral therapy (cART). A retrospective study was carried out using data from the Minimum Basic Data Set (MBDS) and the State Meteorological Agency (AEMET) of Spain. The primary outcome variable was hospital admissions with PTB diagnosis. The environmental risk factors evaluated were season, temperature, humidity, NO_2_, SO_2_, O_3_, PM_10_, and CO. Overall, HIV-infected patients had a lower frequency of PTB-related hospital admissions in summer (22.8%) and autumn (22.4%), but higher values in winter (26.6%) and spring (28.2%). Using a Bayesian temporal model, PTB-related hospital admissions were less frequent in summer-autumn and more abundant in winter-spring during the first years of follow-up. During the later years of follow-up, the seasonal trends continued resulting in the lowest values in autumn and the highest in spring. When considering short-term exposure to environmental risk factors, lower temperatures at 1 week (odds ratio (OR) = 1.03; p = 0.008), 1.5 weeks (OR = 1.03; p<0.001), 2 weeks (OR = 1.04; p<0.001), and 3 weeks (OR = 1.03; p<0.001) prior to PTB admission. In addition, higher concentration of NO_2_ at the time of admission were significantly associated with higher likelihoods of PTB-related hospital admission in HIV-infected patients when 1.5 weeks (OR = 1.1; p = 0.044) and 2 weeks (OR = 1.21; p<0.001) were used as controls. Finally, higher concentration of SO_2_ at 1.5 weeks prior to PTB admission was significantly associated with a higher likelihood of PTB-related hospital admissions (OR = 0.92; p = 0.029). In conclusion, our data suggest an apparent seasonal variation in hospital admissions of HIV-infected patients with a PTB diagnosis (summer/autumn vs. winter/spring), as well as a link to short-term exposure to environmental risk factors, such as temperature and ambient NO_2_ and SO_2_.

## Introduction

Tuberculosis remains one of the world’s deadliest communicable diseases. Despite notable progress in the last decades, tuberculosis is still a public health concern in most of the countries within the WHO European Region [1]. Human immunodeficiency virus (HIV) infection is the most important risk factor for developing tuberculosis in patients already infected with *Mycobacterium tuberculosis* [2,3], and tuberculosis is the most common acquired immunodeficiency syndrome (AIDS)-defining condition worldwide [4]. Globally in 2014, 9.6 million people developed tuberculosis and around 1.5 million died from the disease. At least one-third of people living with HIV worldwide were infected by tuberculosis. There were around 1.2 million new cases of tuberculosis amongst people who were HIV-positive and about four hundred thousand people died of HIV-associated tuberculosis [5]. Among European Union/European Economic Area countries, Spain had one of the highest incidences of AIDS (from 1994 to 2009) and tuberculosis (from 1995 to 2009) [6,7].

*Mycobacterium tuberculosis* is the causative agent of pulmonary tuberculosis (PTB). There are several possible outcomes for a person exposed to *Mycobacterium tuberculosis* bacilli [8]: i) the infection may be immediately destroyed by the host's innate immune response; ii) a proportion of individuals develop active PTB due to an inability to control the initial infection and mount a protective response; iii) in the majority of persons a clinically latent infection will be established, and approximately 5–10% of these will experience reactivation of the infection causing active tuberculosis.

Seasonal variation is a considerable factor in regard to PTB, but the specific effects it has on the epidemic is not entirely clear since several studies have identified incidence peaks in winter, spring, and summer [9–11]. Furthermore, the interpretation of seasonality in relation to PTB risk is complicated due to the large number of factors to consider such as environmental factors (temperature, humidity, sunlight), social factors (crowding and person-to-person contact), and delays in the diagnosis and treatment of tuberculosis particularly in winter [12,13]. These factors seem to affect both primary infection and reoccurrence, although on the latter to a lesser degree. The link between vitamin D deficiency and impaired host defenses against *Mycobacterium tuberculosis* may be a factor since seasonal variability of PTB seems to mirror the seasonal fluctuation of vitamin D levels [13–15]. Vitamin D levels depend on the conversion rate of pro-vitamin D3 to pre-vitamin D3 by sunlight, which is affected by seasonal characteristics (cloud cover, hours of daylight, outdoor activities, exposed skin surface area, etc.), latitude, radiation, etc. [12,15]. Thus, the conversion to pre-vitamin D3 tends to drop in autumn until spring, and the highest conversion rates are found in the summer [12].

PTB is a disease that may be influenced by environmental factors [16]. Hospital admissions in colder months are significantly higher than in warmer months [17,18], however the occurrence of extremely high temperatures during the summer has resulted in a significant increase in the number of tuberculosis cases [19]. Moreover, air pollution is a substantial cause of morbidity and mortality worldwide [20]. Several studies suggest an association between long-term exposure to ambient air pollution and tuberculosis [21–25], and one study found a significant association between short-term ambient air pollution exposure and tuberculosis risk [17]. However, to our knowledge, no epidemiologic studies analyzing environmental factors and PTB incidence in HIV-infected patients has been conducted.

Biologically, environmental factors could be involved in the pathogenesis of tuberculosis through an impact on immune function [16,26,27] and thereby increase susceptibility to developing active PTB. Increased levels of air pollutants have been related to impaired lung function via oxidative stress, which may produce inflammation of the airways, decrease macrophage function, and increase susceptibility to pathogens [16,26]. Moreover, diesel exhaust particles suppress the expression of proinflammatory mediators during tuberculosis infection, inducing a hyporesponsive cellular state, which is a possible mechanism by which air pollutants alter antimicrobial immunity [28]. Also of concern, a weakened immune system caused by HIV infection may promote tuberculosis reactivation [29].

The aim of our study was to evaluate the seasonal variations of hospital admissions with a PTB diagnosis and to determine whether short-term exposure to environmental risk factors (climatological factors and air pollution levels) is related to PTB-related hospital admission in HIV-infected patients in Spain during the era of combined antiretroviral therapy (cART).

## Materials and Methods

### Study population

We performed a retrospective analysis using data of all HIV positive patients aged 16 years and older with a hospital discharge and PTB diagnosis in Spanish hospitals from 1 January 1997 to 31 December 2012. Patients without postal code information in the MBDS were excluded. At hospital discharge, out of 45,427 patients with PTB and postal code in the MBDS, 5,712 were infected with HIV. We did not have postal code for 41% of the records. However, 69% of patients with postal code were distributed in a uniform manner throughout Spain (see S1 Fig).

Data from patients with a diagnosis of PTB were obtained from the Spanish Minimum Basic Data Set (MBDS) provided by the Ministry of Health Social Services and Equality (MSSSI). The MBDS is a clinical and administrative database containing information obtained and recorded at time of hospital discharge, with an estimated coverage of 97.7% of total hospital admissions to public hospitals in Spain [30]. The National Health System (NHS) provides free medical care to 99.5% of the Spanish population, although those persons not covered by the NHS can be attended to at the public hospitals.

The MBDS provided the encrypted patient identification number, sex, date of birth, dates of hospital admission and discharge, patients’ residential postal code, medical institutions providing the services, the diagnosis and procedure codes according to the *International Classification of Diseases*, *9th ed*, *Clinical Modification* (ICD-9-CM), and outcome at discharge. The CMBD includes up to 14 discharge diagnoses and up to 20 procedures performed during the hospital stay. The Spanish MSSSI sets standards for record-keeping and performs periodic audits [30].

### Ethics statement

This study involves the use of patient medical data from the Spanish MBDS, which is hosted by the MSSSI. The MBDS is regulated by law that explains how institutions are required to utilize health-related personal data. As described in detail previously [31], the data were treated with full confidentiality according to Spanish legislation. We requested the databases by filling, signing and sending a questionnaire with a Confidentiality Commitment. The MSSSI evaluated the protocol of our investigation and considered it to meet all ethical aspects according to Spanish legislation. Given the anonymous and mandatory nature of the dataset, it was not necessary to obtain informed consent. Additionally, our study was approved by the Research Ethic Committee (Comité de Ética de la Investigación y de Bienestar Animal) of the Instituto de Salud Carlos III (Madrid, Spain).

### Environmental data

We did not have any data on the exposure levels experienced by individuals. However, exposure to climatic and pollutant factors was obtained as a surrogate using the nearest meteorological station to his or her postal code at the time of hospitalization. Environmental data were provided by the State Meteorological Agency (AEMET) (http://www.aemet.es/). For each station, AEMET provided daily data for temperature, humidity, sulfur dioxide (SO2), carbon monoxide (CO), nitrogen dioxide (NO2), ozone (O3), particulate matter up to 10 μm in size (PM10), and station geolocation (latitude, longitude, and altitude).

### Outcome variables

The outcome variable analyzed in this study was the first PTB-related hospital admission. A hospitalization was defined as a discharge record in the MBDS. The index episode of a patient was defined as the first hospital discharge encoded in MBDS with a PTB diagnosis (ICD-9-CM code 011). Hospital readmissions were not counted as new episode of PTB.

### Statistical analysis

In order to evaluate the seasonal effect on PTB-related hospital admissions, the dates of hospital admission were divided into 4 seasons: spring (March-May), summer (June-August), autumn (September-November) and winter (December-February). A model for seasonal variation was obtained assuming that the sums were independent Gaussian with precision Gamma = (1; 0,001) [32]. The effect was adapted automatically to higher cases in the winter and lower cases in the summer. This specification of the model allows time-varying disease onsets, which is not possible using simple sums of sine and cosine component summer. Seasonal effects were evaluated using a Bayesian model with Poisson distribution [32]. Significance of seasonal effects was calculated based on deviance information criterion.

As described in detail previously [31], a case-crossover design (CCD) was used to evaluate the effect of each environmental factor (temperature, humidity, NO_2_, SO_2_, O_3_, PM_10_, and CO) on the PTB-related hospital admissions. In the CCD, each individual experiencing a health event serves as his or her own reference. In other words, individuals act as their own control [33]. In air pollution epidemiology, CCD is the most suitable study design for studying the effects on health outcomes of varying short-term exposure [34]. In the case of PTB, 4 different short time periods before hospital admission were considered as control periods (1 week, 1.5 week, 2 weeks and 3 weeks), in order to compare the environmental exposure of individual patients at the time of presentation with a PTB-related hospital admission (baseline) [35]. For each time period, we looked at an average value for each environmental factor over a 3-day period (on the day of PTB-related hospital admission and the 2 days immediately prior to hospital admission) in order to mitigate for any single day with an extreme level. Finally, conditional logistic regression (CLR) was used to evaluate the association between environmental factors and hospital admissions. The odds ratio (OR) and its 95% confidence interval were calculated by exact method. In a CCD for each factor, the odds of an event with respect to an increase in the average level of the environmental factor around the date of hospitalization (case at hospital admission) were compared to the change in the factor when PTB hospitalization did not occur (control time at 1 week, 1.5 weeks, 2 weeks, and 3 weeks before hospital admission). In the basic inference, for each case the exposure status during admission (encoded as “1”) and control time (encoded as “0”) are compared, and only subjects with different levels of exposure are informative [34]. In our study, OR values higher than 1 indicate an association of the analyzed factor with higher risk when it is increased at the time of hospital admission or decreased at timepoints before hospital admission; whereas OR values lower than one indicate the analyzed factor is associated with greater risk when it is increased at the control time or decreased at the time of hospital admission. All environmental factors, except temperature, were log-transformed because they varied greatly across dates of measurements. Results of single- and multi- environmental factors model (temperature + humidity + NO_2_ + SO_2_ + O_3_ + PM_10_ + CO) were presented. The multi-environmental factors analysis for air pollution and PTB association was considered because it may account for possible mutual confounding between environmental factors.

Statistical analysis was performed using the R statistical package version 3.1.1 (GNU General Public License) [36]. All tests were two-tailed with p-values <0.05 considered significant.

## Results

### Characteristics of study population

A total of 45,427 patients had a PTB diagnosis in the MBDS between 1997 and 2012, of which 5,712 were HIV positive. Table 1 shows the clinical and epidemiological characteristics of the patients included in the study. The median age was 37.9 years and 80.1% were male. The most frequent comorbidities were mild liver disease (31.9%), chronic pulmonary disease (4.9%), and cancer (3.7%).

**Table 1:** Summary of epidemiological and clinical characteristics of HIV-infected patients with a diagnosis of pulmonary tuberculosis from 1997 to 2012 in Spain.

Description	HIV-infected patients
No. of patients	5,712
Males	4,577 (80.1%)
Age (years)	37.96 (37.74; 38.18)
**Comorbidities**	
Myocardial Infarction	19 (0.3%)
Congestive Heart Failure	21 (0.4%)
Peripheral Vascular Disease	7 (0.1%)
Cerebrovascular Disease	43 (0.8%)
Dementia	30 (0.5%)
Chronic Pulmonary Disease	279 (4.9%)
Connective Tissue Disease-Rheumatic Disease	3 (0.1%)
Peptic Ulcer Disease	18 (0.3%)
Mild Liver Disease	1,824 (31.9%)
Diabetes without complications	87 (1.5%)
Diabetes with complications	2 (0%)
Paraplegia and Hemiplegia	44 (0.8%)
Renal Disease	74 (1.3%)
Cancer	212 (3.7%)
Moderate or Severe Liver Disease	86 (1.5%)
Metastatic Carcinoma	33 (0.6%)

Values are expressed as absolute number (percentage) and mean (95% of confidence interval).

**Abbreviations**: HIV, human immunodeficiency virus.

### Effect of season on PTB-related hospital admission

The annual distribution and seasonal distribution of hospital admissions with PTB diagnoses for the study period are shown in Table 2. The 1997–1999 period had the largest number, but then it decreased during the rest of the study period. Overall, HIV-infected patients had lower frequencies of PTB-related hospital admissions in summer (22.8%) and autumn (22.4%), and higher values in winter (26.6%) and spring (28.2%) (Table 2).

**Table 2:** Summary of annual distribution and seasonal distribution of pulmonary tuberculosis diagnoses between 1997 and 2012 in Spain among HIV-infected patients.

		Seasonal distribution			
Year	Annual	Summer	Autumn	Winter	Spring
**1997**	619 (10.8%)	132 (21.3%)	123 (19.9%)	205 (33.1%)	159 (25.7%)
**1998**	685 (12%)	151 (22%)	121 (17.7%)	209 (30.5%)	204 (29.8%)
**1999**	670 (11.7%)	153 (22.8%)	158 (23.6%)	166 (24.8%)	193 (28.8%)
**2000**	500 (8.8%)	111 (22.2%)	81 (16.2%)	157 (31.4%)	151 (30.2%)
**2001**	446 (7.8%)	96 (21.5%)	100 (22.4%)	118 (26.5%)	132 (29.6%)
**2002**	360 (6.3%)	77 (21.4%)	88 (24.4%)	88 (24.4%)	107 (29.7%)
**2003**	300 (5.3%)	68 (22.7%)	67 (22.3%)	84 (28%)	81 (27%)
**2004**	302 (5.3%)	65 (21.5%)	64 (21.2%)	82 (27.2%)	91 (30.1%)
**2005**	311 (5.4%)	83 (26.7%)	77 (24.8%)	79 (25.4%)	72 (23.2%)
**2006**	190 (3.3%)	52 (27.4%)	44 (23.2%)	36 (18.9%)	58 (30.5%)
**2007**	293 (5.1%)	87 (29.7%)	59 (20.1%)	66 (22.5%)	81 (27.6%)
**2008**	252 (4.4%)	64 (25.4%)	60 (23.8%)	71 (28.2%)	57 (22.6%)
**2009**	284 (5%)	68 (23.9%)	63 (22.2%)	76 (26.8%)	77 (27.1%)
**2010**	110 (1.9%)	30 (27.3%)	26 (23.6%)	25 (22.7%)	29 (26.4%)
**2011**	210 (3.7%)	51 (24.3%)	46 (21.9%)	50 (23.8%)	63 (30%)
**2012**	180 (3.2%)	37 (20.6%)	44 (24.4%)	54 (30%)	45 (25%)
**1997–2012**					
**Median**	5.3%	22.4%	26.6%	28.2%	22.8%
**25th percentile**	4.2%	20.9%	24.3%	26.2%	21.5%
**75th percentile**	8.0%	23.7%	28.6%	29.8%	25.7%
**Interquartile range**	3.8%	2.8%	4.3%	3.6%	4.2%
**Minimum**	1.9%	16.2%	18.9%	22.6%	20.6%
**Maximum**	12.0%	24.8%	33.1%	30.5%	29.7%
**Range**	10.1%	8.6%	14.2%	7.9%	9.1%

**Fig 1:**
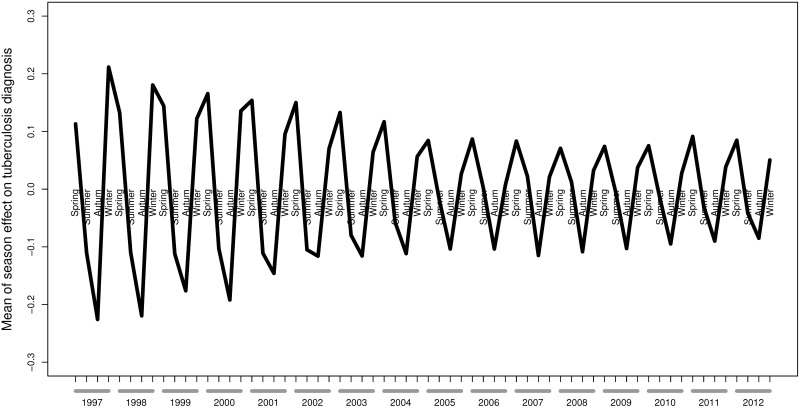
Mean of the seasonal effect on pulmonary tuberculosis diagnosis in HIV-infected patients from Spain between 1997 and 2012.

When seasonal effects were evaluated using a Bayesian model (Fig 1), PTB-related hospital admissions were less frequent in summer-autumn and more abundant in winter-spring during the first years of follow-up, with a transition to the lowest values in autumn and the highest values in spring during the later years of follow-up.

### Effects of short-term exposure to environmental risk factors on PTB-related hospital admissions

When we used a single environmental factor model, we found significant associations of temperature, humidity, NO_2_, SO_2_, O_3_, and PM_10_ with PTB-related hospital admissions (Table 3). Also, we found significant associations of temperature, NO_2_, and SO_2_ with PTB-related hospital admission when we used a multi-environmental factor model (Table 3). Both temperature and NO_2_ concentrations showed significant OR values >1 (Table 3). Thus, lower temperatures at 1 week (OR = 1.03; p = 0.008), 1.5 weeks (OR = 1.03; p<0.001), 2 weeks (OR = 1.04; p<0.001), and 3 weeks (OR = 1.03; p<0.001) prior to PTB admission were significantly associated with higher likelihoods of PTB-related hospital admission in HIV-infected patients, as was higher concentrations of NO_2_ at the time of admission when 1.5 weeks (OR = 1.1; p = 0.044) and 2 weeks (OR = 1.21; p<0.001) were used as controls. Concentrations of SO_2_ showed significant OR values <1 (Table 3); specifically, higher concentrations of SO_2_ at 1.5 weeks prior to PTB admission were significantly associated with higher likelihoods of PTB-related hospital admissions (OR = 0.92; p = 0.029). Note that associations of humidity, O_3_, and PM_10_ with PTB-related hospital admission were lost in the multi-environmental factor model. We did not find any significant association between CO concentrations and risk of PTB hospitalization.

**Table 3:** Summary of associations between short-term exposure of environmental factors and hospital admissions in HIV-infected patients with pulmonary tuberculosis when compared with levels at 1 week, 1.5 weeks, 2 weeks, and 3 weeks before hospital admission.

	Single-factor analysis		Multi-factor analysis	
	OR (95% CI)	p-value	aOR (95% CI)	p-value
**1 week**				
Temperature (°C)	1.03 (1.01; 1.04)	**0.008**	1.03 (1.01; 1.04)	**0.008**
Humidity (%)	0.98 (0.95; 1.01)	0.159	0.98 (0.95; 1.01)	0.15
NO_2_ (μg/m^3^)	1.05 (0.97; 1.15)	0.221	1.09 (0.99; 1.19)	0.086
SO_2_ (μg/m^3^)	0.97 (0.9; 1.04)	0.417	0.95 (0.88; 1.03)	0.224
O_3_ (μg/m^3^)	1 (0.91; 1.09)	0.924	0.97 (0.88; 1.08)	0.620
PM_10_ (μg/m^3^)	0.98 (0.91; 1.06)	0.668	0.98 (0.9; 1.06)	0.586
CO (μg/m^3^)	1 (0.92; 1.09)	0.939	1 (0.92; 1.1)	0.974
**1.5 weeks**				
Temperature (°C)	1.03 (1.01; 1.04)	**0.001**	1.03 (1.02; 1.05)	**<0.001**
Humidity (%)	0.99 (0.96; 1.03)	0.725	1 (0.97; 1.03)	0.827
NO_2_ (μg/m^3^)	1.01 (0.93; 1.1)	0.824	1.1 (1; 1.21)	**0.044**
SO_2_ (μg/m^3^)	0.93 (0.87; 0.99)	**0.025**	0.92 (0.86; 0.99)	**0.029**
O_3_ (μg/m^3^)	1.06 (0.97; 1.17)	0.183	1.02 (0.92; 1.12)	0.754
PM_10_ (μg/m^3^)	0.95 (0.88; 1.02)	0.149	0.95 (0.88; 1.03)	0.197
CO (μg/m^3^)	0.91 (0.84; 0.98)	0.017	0.92 (0.85; 1)	0.063
**2 weeks**				
Temperature (°C)	1.03 (1.02; 1.05)	**<0.001**	1.04 (1.03; 1.05)	**<0.001**
Humidity (%)	0.98 (0.95; 1.01)	0.252	0.98 (0.95; 1.01)	0.141
NO_2_ (μg/m^3^)	1.19 (1.1; 1.29)	**<0.001**	1.21 (1.1; 1.33)	**<0.001**
SO_2_ (μg/m^3^)	1.03 (0.97; 1.1)	0.307	0.98 (0.91; 1.05)	0.500
O_3_ (μg/m^3^)	0.96 (0.88; 1.05)	0.395	0.99 (0.89; 1.09)	0.790
PM_10_ (μg/m^3^)	1.09 (1.01; 1.17)	**0.018**	1.03 (0.95; 1.11)	0.511
CO (μg/m^3^)	1.05 (0.97; 1.13)	0.234	1 (0.92; 1.09)	0.931
**3 weeks**				
Temperature (°C)	1.03 (1.02; 1.04)	**<0.001**	1.03 (1.02; 1.05)	**<0.001**
Humidity (%)	0.97 (0.94; 1)	**0.037**	0.97 (0.94; 1)	0.058
NO_2_ (μg/m^3^)	1 (0.92; 1.08)	0.937	1.07 (0.98; 1.17)	0.149
SO_2_ (μg/m^3^)	0.94 (0.87; 1)	0.050	0.93 (0.87; 1)	0.064
O_3_ (μg/m^3^)	1.1 (1; 1.2)	**0.039**	1.03 (0.93; 1.14)	0.554
PM_10_ (μg/m^3^)	0.97 (0.9; 1.05)	0.467	0.97 (0.9; 1.06)	0.542
CO (μg/m^3^)	0.93 (0.86; 1.01)	0.069	0.96 (0.88; 1.04)	0.313

**Abbreviations**: NO_2_, nitrogen dioxide; SO_2_, sulfur dioxide; O_3_, ozone; PM_10_, particulate matter up to 10 μm in size; CO: carbon monoxide; OR, odds ratio; 95% CI, 95% of confidence interval; HIV, human immunodeficiency virus.

## Discussion

To our knowledge, this is the first report analyzing the influence of seasonality and short-term exposure to climatological factors and air pollution on PTB epidemiology in HIV-infected patients.

In our study, a seasonal effect was observed since the highest frequencies of PTB-related hospital admissions were found in winter-spring. Vitamin D levels may be affected by seasonality [10,11,37], which affects the risk of developing tuberculosis [38]. Sunlight exposure is related to vitamin D production. Specifically, the highest incidence of tuberculosis has been found in areas where ultraviolet exposure is reduced and vitamin D deficiency is more prevalent [11], and low sunlight exposure has been associated with an increased incidence of tuberculosis several weeks later [10,14]. The consensus reached among nutritionists is that the serum 25 (OH) D concentration is quite low or absent above latitude 33°N in winter [39]. Spain lies between 45°N and 55°N, and although the production of pre-vitamin D3 in the skin is higher than in other farther northern European countries, vitamin D levels may also be affected by seasonality [39]. Spain is a European country of the Mediterranean basin with mild winters with colder temperatures, rain and a significant reduction in sunshine exposure, especially in the northern regions, which might contribute to a higher incidence of tuberculosis [40]. Thus, it may be plausible that the reduced exposure to sunshine in the winter and decreased vitamin D production may result in impaired host defense against tuberculosis, and may explain the winter peak (likely new PTB cases) and spring peak (likely PTB reactivations) in our study. Similarly, seasonal changes of neuroendocrine function (glucocorticoid and melatonin levels), which may influence the immune response, have been proposed to contribute to seasonality of infectious diseases [12].

Lower temperatures were a significant risk factor for PTB-related hospital admission in our results. Hospital admissions in colder months are significantly higher than in warmer months [17,18]. Moreover, it has been postulated that climatic conditions during the cold months may facilitate tuberculosis transmission, particularly in overcrowded and poorly ventilated conditions [41,42]. Another factor contributing to seasonal patterns may be other respiratory infections that are more prevalent during the winter months, such as influenza, respiratory syncytial virus or bacterial pneumonia [43]. These infections may impair a person’s immunity during the winter and might lead to the development or reactivation of PTB disease in the spring [43].

Most hospital admissions during the first years of follow-up occurred in the winter, but a transition to peaks of incidence in the spring was found during the later years of follow-up. This seasonal variation might be due to activation of latent *Mycobacterium tuberculosis* infection due to late winter nadirs in vitamin D, but we cannot rule out that it may be due to an increased transmission of tuberculosis due to wintertime indoor crowding. We do not have a clear explanation for this finding, but it might be possible that vitamin D fluctuations explain the seasonality differences in HIV-infected patients. In addition to “classic” risk factors for vitamin D deficiency, cART may affect vitamin D levels [44]. Vitamin D deficiency is a very common disorder in HIV-positive subjects on cART from the Iberian Peninsula [45–47]. In Spain cART was introduced through the national health system in 1996, and since then the percentage of patients on cART has been steadily increasing [48]. Our hypothesis is that this increase in patients on cART could have resulted in a higher percentage of patients with low vitamin D levels, which may be partly responsible. On the other hand, it should not be ruled out that a possible diagnostic delay due to tuberculosis symptoms being similar to other respiratory infections prevalent in winter [49] could result in PTB hospitalizations in the spring.

We also found that higher concentrations of atmospheric pollutants were significant risk factors for PTB-related hospital admission. Specifically, higher concentrations of NO_2_ and SO_2_ prior to hospital admission were a significant risk factor for hospital admissions. However, no association with CO, O_3_, and PM_10_ was found, unlike what has been seen in other studies on the general population [17,21–25,50]. This fact could be due to the limited sample size of our group of HIV-infected patients. Another potential confounding factor may be immune system dysfunction in HIV-infected patients, who have varying degrees of immune system damage according to the stage of their disease and their response to antiretroviral therapy. Of note, among new HIV diagnoses in Spain from 2003–2011, around 30–40% had CD4+ values <200 cells /mm3 in the first test after HIV diagnosis [51,52]. In addition, a large number of HIV patients are intravenous drug users, who usually have limited access to cART and low treatment adherence [53,54].

Tuberculosis is a chronic infection which takes from weeks to months for manifestation of the disease [27]. Previous studies have evaluated the impact of long-term exposure to ambient air pollution on PTB risk [21,24,25], but there is little information about short-term exposure to ambient air pollution and PTB risk [17]. We think short-term exposure to air pollution may play an important role in the exacerbation and pathogenesis of PTB, just as in other chronic respiratory diseases and infections [16]. In fact, short-term exposure to ambient air pollution in adult patients has been associated with lower lung function [55], increases in blood markers of inflammation, decreases in blood markers of coagulation [56], and DNA methylation changes at CpG sites residing in genes involved in inflammation and oxidative stress response [57]. On the other hand, it also reduces early lung immune responses to mycobacteria infection (i.e. it reduces the total immune cell number and causes a significant decline in the recruitment of polymorphonuclear leukocytes as measured in bronchoalveolar lavage fluid, O2 (-) generation, and secretion of TNFα, and IL-6) in an animal model [58].

The ambient air pollution is a persistent public health problem, and millions of people worldwide die each year from causes directly related to air pollution [20,59]. Given the large number of HIV-infected patients with PTB and who are often exposed to high levels of air contaminants, the association between air pollution and PTB may be considered a major public health concern. Thus, interventions to improve air quality could contribute to TB control in patients infected with HIV and in the general population. Moreover, the health services of cities with high levels of air pollution should be alerted to the possible increase in short-term risk of hospital admission due to PTB in the HIV positive population.

Several aspects of the study must be taken into account for a correct interpretation of the results. Firstly, this study had a retrospective design and we had no access to patient clinical data, which would be necessary to fully interpret the impact on PTB-related hospital admission. Secondly, MBDS data are anonymous and it is not possible to identify whether a patient has been hospitalized at different hospitals within the same calendar year. This may have caused a slight overestimation in our results, because we may have considered disease exacerbations or remissions to be new patients. Thirdly, we had no access to detailed information about the microclimate and indoor conditions where each patient was living. Instead of this metric, we defined individual exposure levels based on the closest climatological and air pollution monitoring stations, which can be a good approximation to the actual ambient air conditions for each patient. Fourthly, we had no access to detailed information about indoor air pollution, which is a major risk factor for tuberculosis, particularly from low-income and middle-income countries [60]. However, indoor air pollution is a minor risk factor for tuberculosis in high-income countries [60], and it should not have affected our results.

## Conclusions

In conclusion, our data suggest an apparent seasonal variation in hospital admissions of HIV-infected patients with a PTB diagnosis (summer/autumn vs. winter/spring), as well as a link to short-term exposure to environmental risk factors, such as temperature and ambient NO_2_ and SO_2_. These findings lend further support to the potential role of seasonality and environmental factors in assessing the risk of PTB in patients with HIV infection, and we hope these findings help inform the direction of future research that may contribute to global tuberculosis control.

## Supporting Information

S1 Fig Supplementary Fig 1. Distribution of HIV positive patients aged 16 years and older with a hospital discharge, PTB diagnosis, and postal code in Spanish hospitals from 1 January 1997 to 31 December 2012.
(DOCX)
