# Supplementary material for: Environmental Factors Related to Pulmonary Tuberculosis in HIV-Infected Patients in the Combined Antiretroviral Therapy (cART) Era
Source: PLoS One. 2016 Nov 3;11(11):e0165944. doi: 10.1371/journal.pone.0165944 (PMC5094733; doi:10.1371/journal.pone.0165944)

**Supplementary Figure 1.** Distribution of HIV positive patients aged 16 years and older with a hospital discharge, PTB diagnosis, and postal code in Spanish hospitals from 1 January 1997 to 31 December 2012.


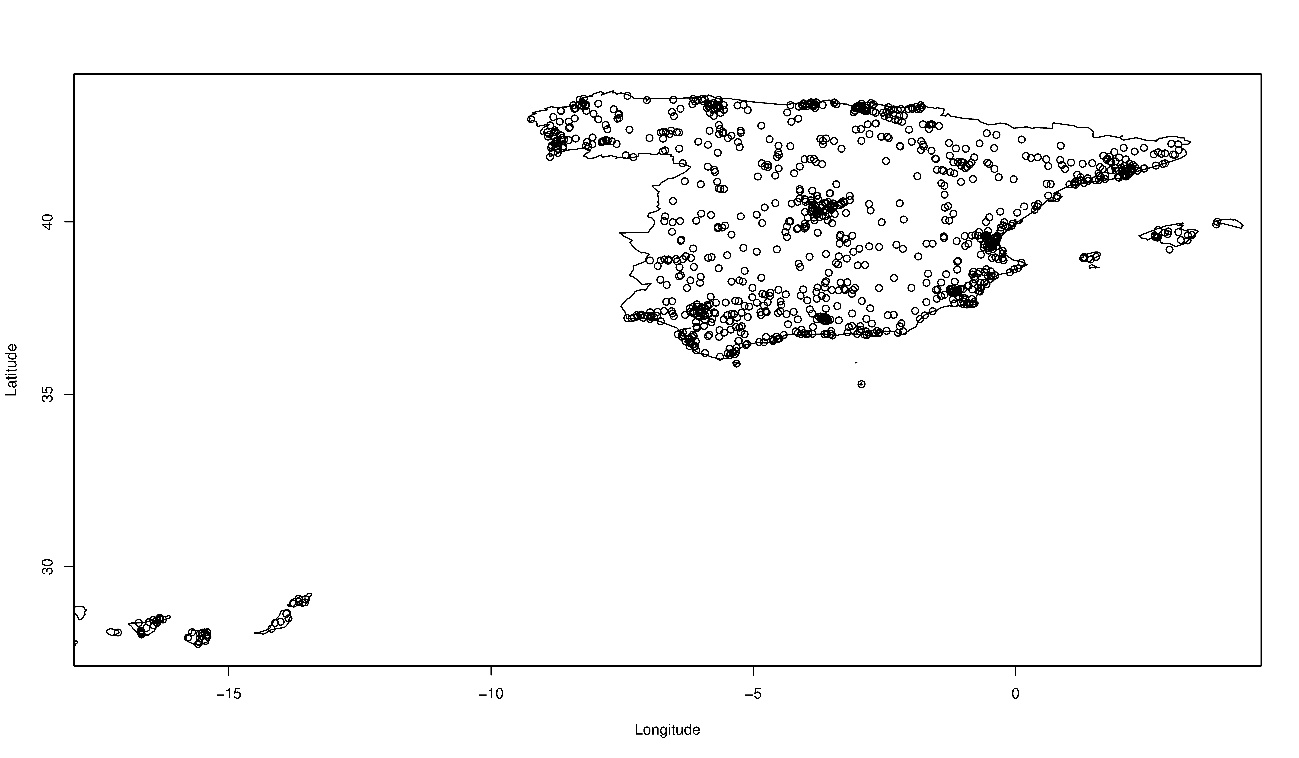

Supplement: S1 Fig — Distribution of HIV positive patients aged 16 years and older with a hospital discharge, PTB diagnosis, and postal code in Spanish hospitals from 1 January 1997 to 31 December 2012. (DOCX) [file pone.0165944.s001.docx]
